# Chemical Composition and Biological Activities of *Gerbera anandria*

**DOI:** 10.3390/molecules19044046

**Published:** 2014-04-02

**Authors:** Fa He, Miao Wang, Minghuan Gao, Min Zhao, Yuhua Bai, Chunjie Zhao

**Affiliations:** 1 Department of Pharmaceutical Analysis, School of Pharmacy, Shenyang Pharmaceutical University, No. 103 Wenhua Road Shenhe District , Shenyang 110016, China; E-Mails: hefa8204@163.com (F.H.); wangmiao_77111@hotmail.com (M.W.); dan_baobaolove@sina.com (M.G.); zm19871224@sina.com (M.Z.); 2 College of Pharmacy, Harbin Medical University, Daqing 163319, China; 3 School of Life Science and Biopharmaceutics, Shenyang Pharmaceutical University, Shenyang 110016, China

**Keywords:** *Gerbera anandria*, coumarins, 8-methoxysmyrindiol, DPPH, cytotoxicity

## Abstract

*Gerbera anandria* (Compositae) was extracted with 75% ethanol and the residue was fractionated using light petroleum, chloroform and ethyl acetate. The constituents of the extracts were separated by column chromatography employing solvents of different polarity. Column chromatography of the light petroleum fraction resulted in the isolation of methyl hexadecanoate, while the chloroform fraction afforded xanthotoxin, 2-hydroxy-6-methylbenzoic acid, 7-hydroxy-1(3*H*)-isobenzofuranone, a mixture of β-sitosterol and stigmasterol, and 8-methoxysmyrindiol and the ethyl acetate fraction gave gerberinside, apigenin-7-*O*-β-d-glucopyranoside and quercetin. A new coumarin, 8-methoxysmyrindiol, was found. The chemical structures of the isolated compounds were established by MS and NMR (HSQC, HMBC). Free radical scavenging and cytotoxic activities of crude extracts and 8-methoxysmyrindiol were further investigated. The ethyl acetate phase exerted the strongest DPPH free radical scavenging activity in comparison to the other fractions. The coumarin 8-methoxysmyrindiol demonstrated cytotoxicity against multiple human cancer cell lines, with the highest potency in HepG2 cells.

## 1. Introduction

*Gerbera anandria* is a plant from the Compositae family, a perennial herb of the *Gerbera* genus, a well-known traditional Chinese medicinal herb and widely grown in China. It is used in Chinese folk medicine to treat coughs, sore throats, as an effective anti-inflammatory, for detoxification, and expelling wind, and is mainly used for rheumatic pains, arthralgia, bruises, sprains, and dysentery. A review of the literature indicates that only a few studies have been conducted on *Gerbera anandria*. Coumarins have been isolated and detected, such as 4-hydroxy-5-methylcoumarin [1], gerberacoumarin [2] and daphnetin 7-methyl ether [3]. Two benzofurans with ethanoyl groups on the pyridine ring were isolated too [4]. Flavonoids such as quercetin were detected in the flowers using high-performance liquid chromatography (HPLC). Also, β-sitosterol and stigmasterol have been reported from this species [5,6]. Coumarins from *Gerbera anandria* were found to have broad spectrum anti-tumor and antibacterial activities *in vitro* and *in vivo* and are considered to be potential new cancer chemoprevention agents. This prompted us to isolate and investigate the chemical composition, as well as the free radical scavenging activity, and *in vitro* cytotoxic activities of the total extract, light petroleum, chloroform and ethyl acetate fractions and some compounds isolated from *Gerbera anandria* in order to evaluate its pharmacological potential.

## 2. Results and Discussion

**Figure 1:**
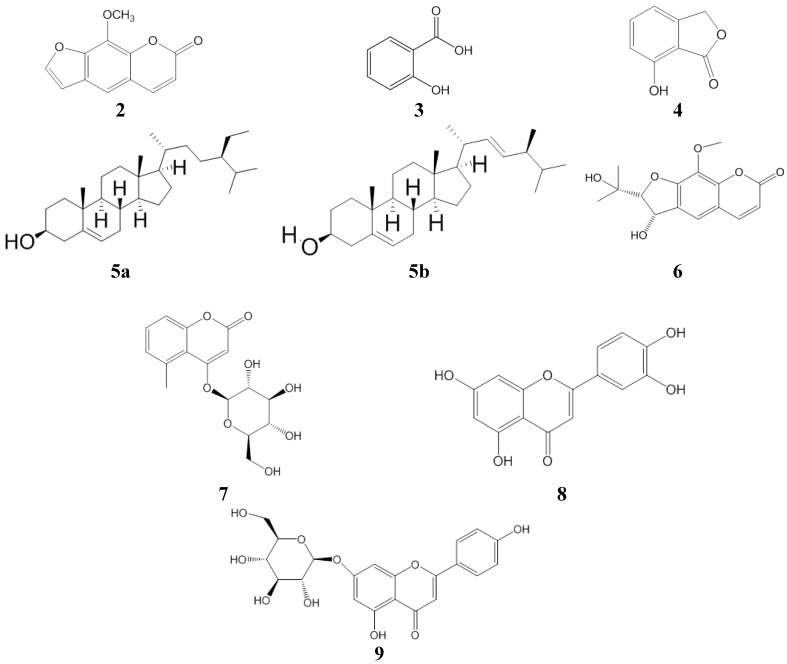
The structures of compounds **2**–**9** isolated from *Gerbera anandria*.

**Figure 2:**
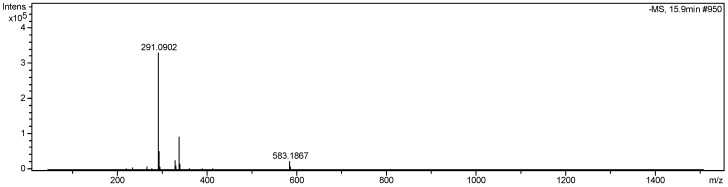
High-resolution mass spectrum of 8-methoxysmyrindiol.

**Figure 3:**
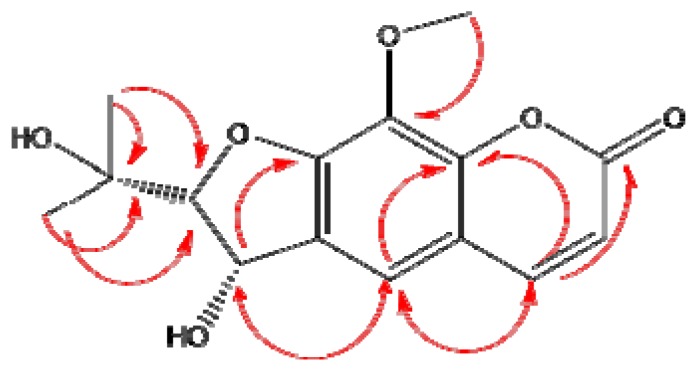
The structure of 8-methoxysmyrindiol supported by the HMBC correlations.

Fractionation of the extracts yielded well-known substances, most of which, however, had not yet been described as constituents of *G. anandria.* Meanwhile a new coumarin, 8-methoxysmyrindiol, was found. The chemical structures of the isolated compounds (Figure 1) were identified using mass spectrometry (MS, Figure 2) as well as nuclear magnetic resonance (1D- and 2D-NMR), including heteronuclear multiple quantum coherence (HSQC), and heteronuclear multiple bond correlation (HMBC, Figure 3).

### 2.1. Antioxidant Activity

The DPPH assay is based on measuring the scavenging ability of antioxidants towards the stable radical 2,2-diphenyl-1-picrylhydrazyl (DPPH) [7]. The free radical DPPH is reduced to the corresponding hydrazine when it reacts with hydrogen donors [8]. The DPPH assay is considered a valid and easy assay to evaluate the antioxidant activity of natural products [9]. The IC_50_ values of different fractions and the standard antioxidant are given in Table 1. The results display the ability of the ethyl acetate fraction to scavenge DPPH, with a stronger scavenging effect than the other fractions. Of the tested compounds, Ascorbic acid used as the positive control, exerted the strongest activity. Most of the isolated compounds from *G*. *anandria* showed lower IC_50_s than the crude extract.

**Table 1:** DPPH scavenging activity of fractions and isolated compounds from *G. anandria*.

Fractions	IC_50_ (μg·mL^−1^)
**Total extract**	30.7 ± 0.4
Petroleum ether fraction	178.8 ± 0.2
Chloroform fraction	66.7 ± 0.3
ethyl acetate fraction	16.0 ± 0.4
**Isolated compounds**	
xanthotoxin	462.1 ± 0.4
2-hydroxy-6-methylbenzoic acid	>500
7-hydroxy-1 (3*H*)-isobenzofuranone	>500
gerberinside	>500
8-methoxysmyrindiol	>500
quercetin	3.5 ± 0.2
**Positive control**	
Ascorbic acid	1.8 ± 0.2
Rutin	2.1 ± 0.3

Data are means ± SE from three independent experiments (*n* = 3).

### 2.2. Cytotoxicity

Table 2 summarizes the IC_50_s of the chloroform fraction and 8-methoxysmyrindiol against various human cancer cell lines. Both show high IC_50_ values in the chemotherapy resistant HepG2 cell line in comparison to the others. Cytotoxicity of chloroform fraction was more pronouncing against HeLa, A375-S2, HT1080 and HL60 cells than 8-methoxysmyrindiol. On the other hand, 8-methoxysmyrindiol was more cytotoxic against MCF-7, HCT11 and A549cells than the chloroform fraction. 

**Table 2:** Cytotoxicity of chloroform fraction and 8-methoxysmyrindiol from *G. anandria* against various human cancer cell lines.

Cell Line	IC_50_ (μg·mL^−1^)	
	Chloroform Fraction 8-Methoxysmyrindiol	
HeLa (human cervical cancer)	60.0 ± 0.2	>100
MCF–7 (human breast cancer)	>100	50.1 ± 0.4
HepG2 (Heptocellular carcinoma)	18.3 ± 0.3	5.3 ± 0.2
HCT116 (human colon cancer)	>100	53.2 ± 0.3
A549 ( human lung adenocarcinoma)	>100	25.1 ± 0.5
A375-S2 (Human melanoma)	33.9 ± 04	>100
HT1080 (Human fibrosarcoma)	40.4 ± 0.2	>100
HL60 (Myeloid leukemia)	28.6 ± 0.3	>100

Data are means ± SE from three independent experiments (*n* = 3).

## 3. Experimental

### 3.1. Plant Material

Fresh *Gerbera anandria* plants were purchased in June 2011 from HeBei Anguo herbal medicine market and air dried. The plant material was botanically identified by Taiming Wei of Harbin Medical University, and a voucher specimen (NO2012809) is deposited in the Herbarium of the Department of Taxonomy, Harbin Medical University, Daqing, China.

### 3.2. Chemicals

2,2 Diphenyl-1-picrylhydrazyl radical (DPPH) was purchased from TCI (Shanghai, China), Fetal bovine serum was purchased from Yuan Heng-Sheng Ma Biotechnology Research Institute (Beijing, China), 3-(4,5 dimethylthiazol-2-yl)-2,5-diphenyl-tetrazolium bromide (MTT), trypsin, dimethyl sulphoxide (DMSO), Cell culture RPMI-1640 media were purchased from Sigma Corporation (St. Louis, MO, USA). Authentic compounds, namely xanthotoxin, 2-hydroxy-6-methylbenzoic acid, 7-hydroxy-1(3*H*)-isobenzofuranone, gerberinside, 8-methoxysmyrindiol, quercetin were isolated by our laboratory extraction; rutin was obtained from Shenyang Pharmaceutical University. In addition, methanol, chloroform, petroleum ether, acetonitrile and other solvents used for extraction, separation and/or detection, which were of analytical grade, were obtained from Yuwang Chemical Factory (Shandong, China). The nuclear magnetic resonance (NMR) spectra were recorded on a Bruker 300 NMR system and a Bruker 600 NMR system (Bruker, Karlsruhe, Germany), CD_3_OD, DMSO-d_6_ and CDCl_3_ were used as solvents. Experimental data were processed using MestRe-C software. Electrospray ionisation mass spectra (ESI-MS) were recorded on a Agilent 1100 LC/MSD Trap/SL (Agilent Teachnology, Santa Clara, CA, USA) under the following conditions: mass scan range, 100–1000 *m/z*; capillary, 3.50 kV; source temp., 325 °C; Drying and nebulising gas was nitrogen.

### 3.3. Extraction Procedure

The whole plants of *Gerbera anandria* (10 kg) were exhaustively extracted with 75% aqueous ethanol (3 × 160 L × 2h) at 80 °C. The ethanolic extract was filtered and then concentrated under vacuum to yield 750 g of a viscous residue. The residue was suspended in water and successively partitioned against light petroleum (b.p. 60–90 °C), chloroform and ethyl acetate. The organic solvents were evaporated under vacuum using rotary evaporator at lower temperature to yield 90 g, 65 g and 60 g of final residues, respectively.

### 3.4. Isolation of the Compounds

#### 3.4.1. Compound **1**

The light petroleum fraction (10 g) redissolved in chloroform was mixed with 10 g silica gel for column chromatography. The dry mixed initial zone was chromatographed on a silica gel column (120 × 2 cm, 200 g) at room temperature. The column was eluted with a gradient using a mixture of chloroform-methanol as mobile phase. Fractions of 200 mL each were collected and column fractions 123–126 eluted at a chloroform-methanol (CHCl_3_-CH_3_OH), (100:1, *v/v*) were collected, evaporated, concentrated under vacuum and monitored by TLC using a mixture of petroleum-acetone (2:1, *v/v*) as a system for TLC development. Spots were detected after spraying with H_2_SO_4_ 10% (*v/v*) in ethanol followed by heating at 105 °C for 5 min, and 15 mg of compound **1** was obtained as white oil from petroleum fraction.

#### 3.4.2. Compounds **2**–**6**

The chloroform fraction (65 g) redissolved in chloroform was mixed with 65 g silica gel for column chromatography and the dry mixed initial zone was chromatographed on a silica gel column (140 × 4 cm, 1 kg) at room temperature, the column was eluted with chloroform-methanol (100:2) as mobile phase, fractions of 5,000 mL were collected, concentrated under vacuum at lower temperature to yield 10 g of final residue. The residue was dissolved in chloroform again and the solution was mixed with 10 g silica gel for column chromatography and the dry mixed initial zone was chromatographed on a silica gel column (120 × 2 cm, 200 g) at room temperature. The column was eluted with a gradient using a mixture of dichloromethane-methanol as mobile phase, Fractions of 200 mL each were collected, evaporated, concentrated under vacuum and monitored by TLC using a mixture of petroleum-acetone (1:1, *v/v*) as a system for TLC development. Spots were detected after spraying with H_2_SO_4_ 10%(*v/v*) in water followed by heating at 105 °C for 5 min, Column fractions 43–176 eluted at a CHCl_2_-CH_3_OH (100:1, *v/v*) were collected, evaporated, fractions having the same R_f_ were pooled and purified either by crystallization or by preparative TLC or by Sephadex LH-20 gel column chromatography, compounds **2**–**5** were obtained: Fractions 47–62 afforded 50 mg of compound **2** as white filamentous crystals from chloroform, giving green fluorescence at 365 nm with the R_f_ 0.75; fractions 68–73 afforded 8 mg of compound **3** as needle-shaped crystals from chloroform, that gave blue fluorescence at 254 nm with the R_f_ 0.15; fractions 76–80 afforded 5 mg of compound **4** as a white amorphous powder from chloroform that gave purple fluorescence at 365 nm with the R_f_ 0.52. Fractions 124–130 afforded 20 mg of compound **5** as white needle-shaped crystals, showing a purple spot after spraying and heating. The collected fractions 184–198 eluted at a CHCl_2_-CH_3_OH, (100:2, *v/v*) afforded 20 mg of compound **6** as white needles crystals from methanol by preparative TLC, and compound 6 also showed blue fluorescence at 365 nm with the R_f_ 0.23.

#### 3.4.3. Compounds **7**–**9**

The ethyl acetate fraction (60 g) redissolved in chloroform was mixed with 60 g silica gel for column chromatography and the dry mixed initial zone was chromatographed on a silica gel column (140 × 4 cm, 1 kg) at room temperature, the column was eluted with chloroform-methanol (100:10) as mobile phase, fractions of 6,000 mL were collected, concentrated under vacuum at lower temperature to yield 8 g of final residue; Then the residue was dissolved in chloroform again and the solution was mixed with 8 g silica gel for column chromatography and the dry mixed initial zone was chromatographed on a silica gel column (120 × 2 cm, 160 g) at room temperature. The column was eluted with a gradient using a mixture of dichloromethane-methanol as mobile phase, Fractions of 200 mL each were collected, concentrated under vacuum and monitored by TLC using chloroform-methanol (5:2, *v/v*) as developing system. The TLC was visualised under UV and 3% AlCl_3_ (*v/v*) in ethanol spray reagent. Column fractions 47–213 eluted at a CHCl_2_-CH_3_OH (100:5, *v/v*) were collected, evaporated and fractions having the same R_f_ were pooled and purified either by crystallisation or by preparative TLC or by Sephadex LH-20 gel column chromatography to afford compounds **7**–**9**. Fractions 47–62 afforded 30 mg of compound **7** as white needle-shaped crystals from methanol, that gave blue fluorescence at 365 nm with R_f_ 0.25; fractions 83–96 afforded 12 mg of compound **8** as yellow needle-shaped crystals from methanol, that showed yellow-green fluorescence at 365 nm; fractions 134–153 afforded 6 mg of compound **9** as yellow needle-shaped crystals from methanol, that displayed yellow-green fluorescence at 365 nm with R_f_ 0.55; 

### 3.5. Structure Identification

*Methyl hexadecanoate* (**1**). Yield: 50 mg; C_17_H_34_O_2_, White grease, m.p.: 28–30 °C. ESI-MS *m/z* 255.1 [M–CH_3_]^−^ ; ^1^H-NMR (300 Hz, CDCl_3_), δ (ppm): 0.88 (3H, t, 16-H), 1.26 (24H, s, 4-15-H), 1.62 (2H, t, *J =* 7.2 Hz, 3-H), 2.30 (2H, q, 2-H), 3.66 (2H, s, 1'-H). ^13^C-NMR (CDCl_3_), δ (ppm): 174.30 (C-1), 51.42 (C-1'), 34.09 (C-2'), 31.92 (C-14), 29.69 (C-7, C-11), 29.59 (C-12), 29.45 (C-13), 29.36 (C-6), 29.25 (C-5), 29.14 (C-4), 24.94 (C-3), 22.68 (C-15), 14.11 (C-16). Compound **1** was identified as methyl hexadecanoate from these spectral data and physical properties [10].

*Xanthotoxin* (**2**). Yield: 50 mg; C_12_H_8_O_4_, White filamentous crystals, m.p.: 143–148 °C. ESI-MS *m/z* 216.9 [M]^+^, 239.4 [M+Na]^+^, 455.4 [2M+Na]+. ^1^H-NMR (300 Hz, CDCl_3_), δ (ppm): 7.76 (1H, d, *J =* 9.6 Hz, 4-H) , 7.69 (1H, d, *J =* 2.1 Hz, 2'-H), 7.35 (1H, s, 5-H), 6.82 (1H, d, *J =* 2.4 Hz, 3'-H), 6.37 (1H, d, *J =* 9.6 Hz, 3-H), 4.30 (3H, s, 8-OCH_3_). ^13^C-NMR (CDCl_3_), δ (ppm): 160.43 (C-2), 147.70 (C-7), 146.63 (C-2'), 144.30 (C-4), 143.02 (C-9), 132.82 (C-8), 126.12 (C-6), 116.50 (C-10), 114.77 (C-3), 112.89 (C-5), 106.72 (C-3'), 61.33 (OCH_3_). Compound **2** was identified as xanthotoxin from these spectral data and physical properties [11].

*2-Hydroxy-6-methylbenzoic acid* (**3**). Yield: 8 mg; C_8_H_8_O_3_, White needle-shaped crystals , rendered blue fluorescence (254 nm), ESI-MS *m/z* 151.4 [M–H]^−^. ^1^H-NMR (300 Hz, CDCl_3_), δ (ppm): 11.06 (1H, s, 1-COOH, 2-OH) ,7.34 (1H, t, *J =* 7.5 Hz, 8.1 Hz, 4-H), 6.87 (1H, d, *J =* 8.4 Hz, 5-H), 6.77 (1H, d, *J =* 7.5 Hz, 6-H), 2.63 (3H, s, 3-CH_3_). ^13^C-NMR (CDCl_3_), δ (ppm): 174.66 (COOH), 163.35 (C-2), 142.44 (C-6), 134.58 (C-4), 123.50 (C-5), 115.89 (C-3), 114.67 (C-1), 23.51 (CH_3_). Compound **3** was identified as 2-hydroxy-6-methylbenzoic acid from these spectral data and physical properties [12].

*7-Hydroxy-1(3H)-isobenzofuranone* (**4**). Yield: 5 mg; C_8_H_6_O_3_, White amorphous powder, rendered purple fluorescence (365 nm), m.p.: 135–136 °C. ESI-MS *m/z* 148.9 [M–H]^−^. ^1^H-NMR (600 Hz, CDCl_3_), δ (ppm): 7.76 (1H,s,7-OH), 7.57 (1H, t, *J =* 7.8 Hz, 5-H), 6.98 (1H, d, *J =* 7.8Hz, 4-H), 6.94 (1H, d, *J =* 7.8Hz, 6-H), 5.33 (2H, s, 3-H), ^13^C-NMR (CDCl_3_), δ (ppm): 172.59 (C-1), 156.63 (C-7a), 146.72 (C-7), 136.97 (C-5), 115.32 (C-4), 113.37 (C-6), 110.93 (C-3a), 70.62 (C-3). The structure of was supported by the HMBC correlations of 7-OH(δ7.76) with C-6,C-7; H-5,(δ7.57 ) with C-3a,C-7; H-4,(δ6.98) with C-3, C-7a, C-6; H-6 (δ6.94 ) with C-7, C-4, C-7a, C-1; H2-3 (δ5.33) with C-3a, C-1, C-5, C-7a, C-4, C-6, C-7. Compound **4** was identified as 7-hydroxy-1(3*H*)-isobenzofuranone from these spectral data and physical properties [13]. 

*Compound* **5**. White needle-shaped crystals, ^1^H-NMR (300 MHz, CDCl_3_), δ (ppm): 3.5 (1H, tt, *J =* 4.5, 11.1 Hz, H-3), 5.4 (1H, br. s, H-6), 0.7 (3H, s, H-18), 1.0 (3H, s, H-19), 0.9 (3H, d, *J =* 6.6 Hz, H-21), 5.2 (1H, dd, *J =* 8.7, 15.0 Hz, H-22), 5.0 (1H, dd, *J =* 8.7, 15.2 Hz, H-23), 0.8 (3H, d, *J =* 6.8 Hz, H-26), 0.8 (3H, d, *J =* 7.4 Hz, H-27), 0.8 (3H, t, *J =* 7.6 Hz, H-29); ^13^C-NMR (125 MHz, CDCl_3_), δ (ppm): 37.3 (C-1), 31.7 (C-2), 71.8 (C-3), 42.3 (C-4), 140.8 (C-5), 121.7 (C-6), 31.9 (C-7), 31.9 (C-8), 50.2 (C-9), 36.5 (C-10), 21.1 (C-11), 39.8 (C-12),42.3 (C-13), 56.8 (C-14), 24.3 (C-15), 28.9 (C-16), 55.9 (C-17), 12.0 (C-18), 19.4 (C-19), 36.5 (C-20), 19.4 (C-21), 33.9, 138.3 (C-22), 26.1, 129.3 (C-23), 45.8, 51.2 (C-24), 29.2, 31.7 (C-25), 19.4 (C-26),19.8 (C-27), 23.1, 24.3 (C-28), 12.0, 12.2 (C-29). This compounds was identified as a mixture of β-sitosterol (**5a**) and stigmasterol (**5b**) from these spectral data and physical properties [14,15].

*8-Methoxysmyrindiol* (**6**). Yield: 20 mg; C_15_H_16_O_6_, white granules, HRESIMS (Figure 2) *m/z* 291.0902 [M–H]^−^ , 336.9002 [M+COOH]^−^, 583.1857 [2M–H]^−^. ^1^H-NMR (600 Hz, CDCl_3_), δ (ppm): 7.87 (1H, d, *J =* 9.6Hz, 4-H ), 7.31 (1H, s, 5-H), 6.24 (1H, d, *J =* 9.6Hz, 3-H), 5.37 (1H,d, *J =* 6Hz, 3'-H), 4.42 (1H,d, *J =* 6Hz, 2'-H), 4.05 (3H, s, 8-OCH3), 1.51 (3H, s, 2''-H), 1.47 (3H, s, 3''-H). ^13^C-NMR (CDCl_3_), δ (ppm): 162.89 (C-2), 155.22 (C-7), 149.28 (C-8a), 146.412 (C-4), 132.87 (C-8), 130.77 (C-6), 119.92 (C-5), 115.66 (C-4a), 113.03 (C-3) , 93.38 (C-2') ,73.14 (C-1''), 72.52 (C-3') , 61.30 (8-OCH3) , 27.12 (C-3''), 26.52 (C-2''). The structure was supported by the HMBC correlations of H-4 (δ7.87) with C-8a, C-2, C-5; H-5 (δ7.31) with C-3', C-4, C-8a; H-3 (δ6.24) with C-4a; H-3'(δ5.37) with C-5, C-7, C-6; OCH3 (δ4.05) with C-8; H-2''(δ1.51) and H-3''(δ1.47) with C-1'',C-2' (Figure 2). Compound **6** was identified as 8-methoxysmyrindiol [16].

*Gerberinside* (**7**). Yield: 30 mg; C_16_H_18_O_8_, white needle-shaped crystals , ESI-MS *m/z* 336.9 [M–H]^−^, 383.0 [M+Cl] ^ −^, 400.0 [M+HCOOH]^−^. ^1^H-NMR (600 Hz, CDCl_3_), δ (ppm): 7.47 (1H, t, *J =* 7.8, 7.8Hz, 7-H), 7.19 (1H, d, *J =* 8.4 Hz,8-H) , 7.14 (1H, d, *J =* 7.8Hz, 6-H), 5.98 (1H, s, 3-H), 5.23 (1H, d, *J =* 7.8Hz, 1'-H), 3.91 (1H, dd, *J =* 2.4, 2.4 Hz, 6'-H), 3.72 (1H, dd, *J =* 5.4, 5.4 Hz, 6'-H), 3.60 (1H, t, *J =* 7.8, 1.2 Hz, 2'-H), 3.55 (1H, m, 3'-H), 3.50 (1H, t, *J =* 9.6, 9.0 Hz, 3'-H ), 3.43 (1H, t, *J =* 9.6, 9.0 Hz, 4'-H ), 2.77 (3H, s, 5-CH_3_), ^13^C-NMR (CDCl_3_), δ (ppm): 168.99 (C-4), 164.84 (C-2), 156.12 (C-9), 138.99 (C-5), 133.14 (C-7), 129.15 (C-6), 116.05 (C-8),115.41 (C-10), 101.29 (C-1'), 94.11 (C-3),78.66 (C-3'), 78.31 (C-5'), 74.59 (C-2') , 70.88 (C-4'), 62.21 (C-6') , 23.81 (5-CH3). The structure of was supported by the HMBC correlations of H-1'(δ5.23) with C-4, C-2',C-5 and H-6'(δ3.72) with C-4', C-5'. Compound **7** was identified as gerberinside [17].

*Quercetin* (**8**). C_15_H_10_O_7_, yellow needle-shaped crystals, with the same R_f_ values of quercetin using three different TLC developing systems.

*Apigenin-7-O-β-D-glucopyranoside* (**9**). C_21_H_20_O_10_, yellow needle-shaped crystals ^1^H-NMR (300MHz, DMSO-*d*_6_), δ (ppm): 12.98 (1H, s, 5-OH), 10.41 (1H, s, 4'-OH), 7.95 (2H, d, *J =* 8.7Hz, H-2',6'), 6.94 (2H, d, *J =* 8.7Hz, H-3',5'), 6.88 (1H, d, *J =* 1.2Hz, H-8),6.81 (1H, s, H-3), 6.46 (1H ,d, *J =* 1.2Hz, H-6), 5.08 (1H, d, *J* *=* 6.6Hz, H-1''). ^13^C-NMR (DMSO-d_6_), δ (ppm): 182.15 (C-4), 164.38 (C-2), 163.08 (C-7), 161.51 (C-4'), 161.25 (C-5), 157.08 (C-9), 128.89 (C-6'), 128.62 (C-2'), 121.15 (C-1'), 116.19 (C-3'), 116.19 (C-5'), 105.46 (C-10), 103.19 (C-3), 99.64 (C-6), 99.64 (C-1''), 92.9 (C-8), 77.30 (C-3''), 76.57 (C-5''), 73.21 (C-6''), 69.64 (C-4''), 60.50 (C-7''). Compound **9** was identified as apigenin-7-O-β-d-glucopyranoside from these spectral data and physical properties [18]. 

### 3.6. Antioxidant Activity

Stock solutions of the crude methanol extract and fractions (light petroleum, chloroform, ethyl acetate) were prepared by dissolving 10 mg in 1 mL methanol. Serial dilution was done from each stock solutions and 1mL from each dilution was tested individually by adding to 2 mL of 0.05 mg·mL^−1^ 2,2-diphenyl-1-picrylhydrazyl (DPPH). The mixtures were vigorously shaken and allowed to stand in the dark for 15 min at room temperature. Absorbance was measured at 515 nm against methanol as blank. The inhibition rate of each extract against DPPH radical was calculated according to the following formula:

Y = [1−(Ai − Aj)/A0] × 100%

**A_i_**: The absorbance of adding the sample solutions; 

**A_0_**: The absorbance of adding 1mL methanol instead of the sample solutions; 

**A_j_**: The absorbance of adding 2 mL methanol instead of the DPPH solutions.

The curve was drawn with the clearance *Y* as ordinate and the mass concentration *m* as abscissa. The free radical scavenging activity, expressed as IC_50_ (μg·mL^−1^), was compared with standard antioxidants such as ascorbic acid.

### 3.7. Cytotoxicity

#### 3.7.1. Cell Culture

HeLa, HepG2, HT-1080, HCT116, A375-S2, MCF-7, A549, HL60 cells were purchased from American Type Culture Collection (ATCC, Rockville, MD, USA) and maintained in RPMI-1640 (2% glutamine) supplemented with 10% foetal bovine serum (FBS), Cells were grown in a humidified atmosphere of 5% CO_2_ at 37 °C.

#### 3.7.2. MTT Assay

##### 3.7.2.1. Adherent Cells

Sensitivity of the cells to drugs was determined in triplicate using the MTT cell viability assay [19,20]. Exponentially growing adherent tumor cell lines (5 × 10^4^/mL ) were seeded in a 96-well plate (Corning, Corning, NY, USA) after trypsinization [21], 100 μL each hole. After incubation for 24 h the cells were incubated with 100 μL fresh medium containing various concentrations of compounds at 37 °C for 48 h. The medium was removed and cells were incubated with 100 μL fresh medium containing 0.5 mg·mL^−1^ MTT. During incubation for further 4 h, MTT is reduced by mitochondrial dehydrogenases of viable cells to a purple formazan product. The medium was discarded and 150 μL DMSO was added. The plates were shaken for 10 min at room temperature, and the spectrophotometric absorbance was monitored at 492 nm using Tecan-ELISA plate reader.

##### 3.7.2.2. Suspension Cells 

Exponentially growing cell lines (2 × 10^5^/mL)were seeded in a 96-well plate, 50 μL Each hole (Corning). After incubation for 24 h the cells were incubated with 50 μL fresh medium containing various concentrations of compounds at 37 °C for 48 h. The medium was removed and cells were incubated with 10 μL fresh medium containing 5 mg·mL^−1^ MTT. During incubation for further 4 h, MTT is reduced by mitochondrial dehydrogenases of viable cells to a purple formazan product. The medium was discarded and formazan crystal dissolved in 100 μL triple solution (SDS 10 g, 10M HCl 0.1 mL, isobutanol 5 mL, diluted with distilled water to 100 mL). After incubation for further 12 h at 37 °C, the spectrophotometric absorbance was monitored at 492 nm using Tecan-ELISA plate reader.

##### 3.7.3. Evaluation of Results

All experiments were repeated at least three times. Results are reported as means ± SE. The IC50 was determined as the drug concentration which resulted in a 50% reduction in cell viability or inhibition of the biological activity. The inhibition rate (Y) was calculated according to the following formula:

Y = (A_0_ – A_i_)/A_0_ × 100%

**A_0_**: The absorbance of Negative control; **A_i_**: The absorbance of adding the sample solutions.

## 4. Conclusions

A new coumarin 8-methoxysmyrindiol and eight other compounds were isolated from the 75% aqueous ethanol extract of the whole plants of *Gerbera anandria*. The chemical structures of the isolated compounds were established by MS and NMR (APT, HSQC and HMBC). Free radical scavenging and cytotoxic activities of crude extracts and 8-methoxysmyrindiol were further investigated. The ethyl acetate phase exerted the strongest DPPH free radical scavenging activity in comparison to other fractions, this may be due to the fact that the majority of the flavonoids are normally present in the ethyl acetate phase. The coumarin 8-methoxysmyrindiol demonstrated cytotoxicity against multiple human cancer cell lines, with the highest potency in HepG2 cells.

## Supplementary Materials

Supplementary materials can be accessed at: http://www.mdpi.com/1420-3049/19/4/4046/s1.
