# Supplementary material for: Chemical Composition and Biological Activities of Gerbera anandria
Source: Molecules. 2014 Apr 2;19(4):4046–57. doi: 10.3390/molecules19044046 (PMC6271908; doi:10.3390/molecules19044046)
Supplement: Supplementary file 1 [file molecules-19-04046-s001.pdf]

Supplementary File

A new coumarin 8-methoxylsmyrindiolc and eight other compounds were isolated from the 75% aqueous ethanol extract of the whole plants of Gerbera anandria. The structure of 8-methoxysmyrindiol (Figure S1) was supported by the HMBC correlations of H-4(δ7.87) with C-8a, C-2, C-5; H-5(δ7.31) with C-3', C-4, C-8a; H-3(δ6.24) with C-4a; H-3'(δ5.37) with C-5, C-7, C-6; OCH3(δ4.05) with C-8; H-2''(δ1.51) and H-3''(δ1.47) with C-1'',C-2'.

Figure S1. The structure of 8-methoxysmyrindiol supported by the HMBC correlations.

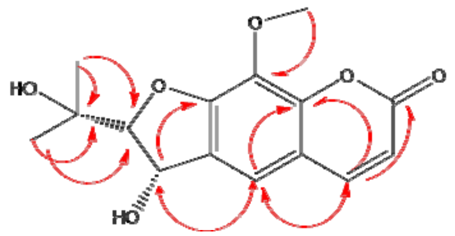

Free radical scavenging and cytotoxic activities of crude extracts and 8-methoxysmyrindiol were further investigated .The ethyl acetate phase exerted the strongest DPPH free radical scavenging activity in comparison to other fractions. Table S1 summarises IC_{50}s of chloroform fraction and 8-methoxysmyrindiol against various human cancer cell lines. Coumarin 8-methoxysmyrindiol demonstrated cytotoxicity against multiple human cancer cell lines, with the highest potency in HepG2 cells.

Table S1. Cytotoxicity of chloroform fraction and 8-methoxysmyrindiol from *G. anandria* against various human cancer cell lines.

**Cell Line**	**IC_{50} (μg·mL^{-1})**	
	**Chloroform Fraction**	**8-Methoxysmyrindiol**
HeLa (human cervical cancer)	60.0 ± 0.2	>100
MCF–7 (human breast cancer)	>100	50.1 ± 0.4
HepG2 (Heptocellular carcinoma)	18.3 ± 0.3	5.3 ± 0.2
HCT116 (human colon cancer)	>100	53.2 ± 0.3
A549 (human lung adenocarcinoma)	>100	25.1 ± 0.5
A375-S2 (Human melanoma)	33.9 ± 04	>100
HT1080 (Human fibrosarcoma)	40.4 ± 0.2	>100
HL60 (Myeloid leukemia)	28.6 ± 0.3	>100
